# Quantum and electrochemical interplays in hydrogenated graphene

**DOI:** 10.1038/s41467-018-03026-0

**Published:** 2018-02-23

**Authors:** Lin Jiang, Wangyang Fu, Yuvraj Y. Birdja, Marc T. M. Koper, Grégory F. Schneider

**Affiliations:** Leiden University, Faculty of Science, Leiden Institute of Chemistry, Einsteinweg 55, Leiden, 2333CC The Netherlands

## Abstract

The design of electrochemically gated graphene field-effect transistors for detecting charged species in real time, greatly depends on our ability to understand and maintain a low level of electrochemical current. Here, we exploit the interplay between the electrical in-plane transport and the electrochemical activity of graphene. We found that the addition of one H-*sp*^3^ defect per hundred thousand carbon atoms reduces the electron transfer rate of the graphene basal plane by more than five times while preserving its excellent carrier mobility. Remarkably, the quantum capacitance provides insight into the changes of the electronic structure of graphene upon hydrogenation, which predicts well the suppression of the electrochemical activity based on the non-adiabatic theory of electron transfer. Thus, our work unravels the interplay between the quantum transport and electrochemical kinetics of graphene and suggests hydrogenated graphene as a potent material for sensing applications with performances going beyond previously reported graphene transistor-based sensors.

## Abstract (web summary)

Electrochemically-gated graphene field-effect transistors show promise for sensing of charged species in real time. Here, the authors leverage the interplay between electrical in-plane transport and electrochemical activity to explore the sensing performance of hydrogenated graphene.

## Introduction

Graphene is unique among other solid-state materials in that all carbon atoms are located on the surface, making the graphene surface highly sensitive for the detection of changes in the environment. Particularly, the concept of electrochemically gated graphene field-effect transistor (GFET) enables the label-free detection of charged molecules on a small footprint upon their bindings at/near the graphene surface:^1,2^ a binding event modulates the electrical current in the graphene channel through the local variation of the electric field^3,4^. The creation of practical electrochemically gated GFETs for detecting charged species, however, greatly depends on our ability to understand and maintain a low level of electrochemical current. Specifically, the electrochemical current roots on the current flowing between the graphene channel and redox active molecules in the solution phase.

Complementary to GFET sensors, the electrochemical current towards a redox probe in solution has been widely studied and is at the basis of graphene electrochemical (GEC) sensors. Previous studies have revealed that the electrochemical activity is largely sensitive to the intrinsic chemical structure of the graphene basal plane^5–8^. Among the multiple approaches used to chemically modify graphene, for example, post-growth chemical modifications using various oxidative reactions^9–11^ are effective routes to incorporate oxygen atoms, although at the cost of a poor control over the resulting functional groups (i.e., epoxy, carbonyl, carboxylic acid, alcohol, all at the same time). Particularly, hydrogenated or fluorinated graphene endows a large range of possibilities to progressively tweak graphene with *sp*^3^ defects without significantly pinning the lattice integrity or breaking the resilient basal plane C–C bonds^12–15^.

Here, a low density of H-*sp*^3^ defects are introduced into monolayer graphene using a hydrogen plasma. We found that only 1 s of plasma treatment is able to render a pristine graphene surface (with few H-*sp*^3^ defects) from the as-grown graphene (referred as untreated graphene) by removing the adsorbed hydrocarbons at the surface, as manifested by the dramatic boost in the electron transfer rate. Importantly, further addition of only one H-*sp*^3^ defect per hundred thousand carbon atoms (more than 1 s of hydrogen plasma), allows us to substantially reduce the electron transfer rate of hydrogenated graphene (H-graphene) compared to pristine graphene. Remarkably, we successfully correlated the degradation of the electrochemical kinetics of the graphene basal plane with the density of states (DOS) by tuning the density of H-*sp*^3^ defects. Although the H-*sp*^3^ termination could contribute to a higher electrochemical activity, the electronic structure (DOS) in graphene plays an even more decisive role in the rate of electron transfer between graphene and redox probes for a low defect density, indicating a non-adiabatic transfer process on the graphene basal plane.

## Results

### Raman characterization

**Fig. 1:**
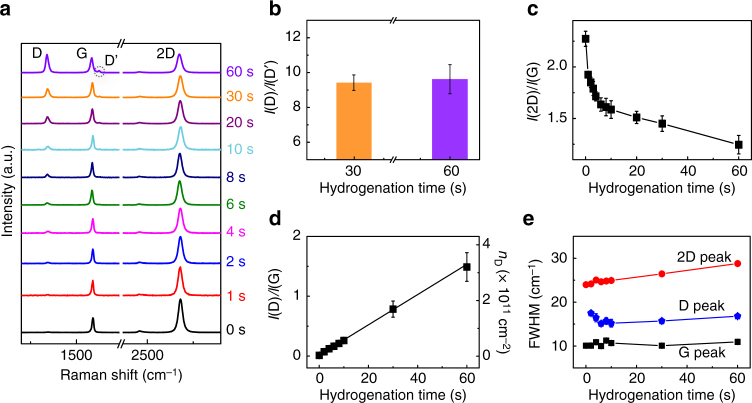
Raman characterization of hydrogenated graphene. **a** Averaged Raman spectra of CVD graphene on a Si/SiO_2_ substrate after 0-60 s of H_2_ plasma (10 W, 1.0 mbar). **b** The intensity ratio *I*(D)/*I*(D′) after 30 s and 60 s of hydrogenation. **c** The intensity ratio *I*(2D)/*I*(G) for hydrogenation times ranging from 0 to 60 s. **d** The intensity ratio *I*(D)/*I*(G) and the derived defect density *n*_D_, plotted vs the hydrogenation time. The error bars include results from both exfoliated and CVD graphene. **e** The FWHM of the 2D, G, and D peaks vs the hydrogenation time. The spectra are recorded using a 2.33 eV (532 nm) laser excitation. The error bars in **b**–**e** are the standard deviation of experimental values

To determine the density and the nature of the defects induced by hydrogen radicals, we conducted Raman spectroscopy (Fig. 1a) and mapping (Supplementary Figure 1a) on graphene prepared by chemical vapor deposition (CVD). The similarities between the Raman spectra for both CVD and exfoliated graphene (Supplementary Figure 2) indicate that the defects induced by the H_2_ plasma—particularly the defect density *n*_D_—are respectively equivalent. Importantly, the D peak at ~1340 cm^−1^, due to single phonon intervalley scattering events, is caused by the apparition of H-*sp*^3^ defects^16^. After a hydrogenation time of 10 s, a D′ peak at 1620 cm^−1^ appears in the Raman spectrum as a shoulder of the G peak. The D′ peak also associates with H-*sp*^3^ defects^17^. The values determined for *I*(D)/*I*(D′) (~10) after 30 s and 60 s of hydrogenation are consistent with a previous report and confirm the *sp*^3^ nature of hydrogenated defects (Fig. 1b)^18^. Meanwhile, the intensity ratio *I*(2D)/*I*(G), a sensitive parameter to graphene doping, decreases continuously from 2.2 to 1.3 upon extended hydrogenation (Fig. 1c)^19,20^.

Derived from the *I*(D)/*I*(G) ratio (a quantitative indicator of point defects in graphene samples)^21^, the defect density *n*_D_ increases linearly with the hydrogenation time from *n*_D_ = (0.2 ± 0.3) × 10^10^ cm^−2^ at 0 s (untreated graphene) to *n*_D_ = (3.2 ± 0.7) × 10^11^ cm^−2^ at 60 s, corresponding to a decrease in the average distance between defect sites (*L*_D_) from 122.6 nm to 10.0 nm (Fig. 1d, see Supplementary Note 1 for the calculation of *n*_D_ and *L*_D_). Notably, the Raman mapping (D peak intensity) in Supplementary Figure 1b on exfoliated graphene flakes (which contains minimal native defects, except for edges), confirms the uniform defect distribution upon hydrogenation. Other surface characterizations including scanning electron microscopy (SEM) and atomic force microscopy (AFM) (Supplementary Figure 3) further revealed the non-cracked and preserved lattice of H-graphene. Moreover, the low defect densities are also in agreement with the relatively small variations observed for the full-width at half-maximum (FWHM) of the D and G peaks (2–5 cm^−1^, Fig. 1e)^22^. In addition, the peak broadening as hydrogenation proceeds is mainly due to the shortened lifetime of phonons caused by increasing amounts of defects^21,22^.

### Electrical transport measurement

**Fig. 2:**
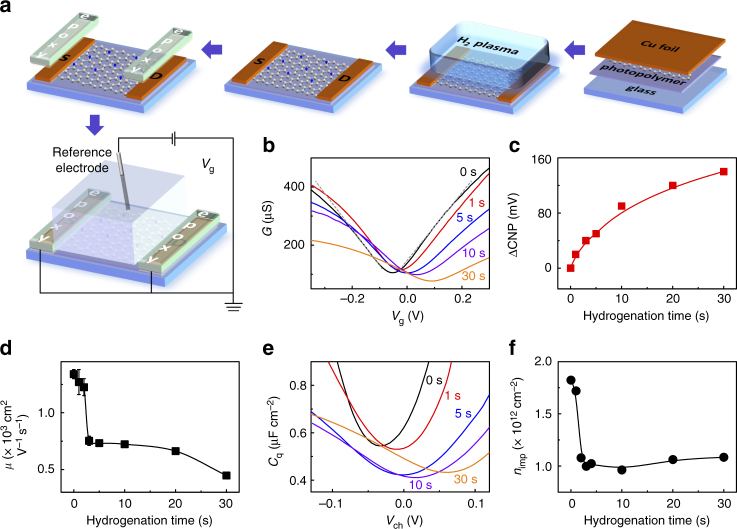
Transport characteristic and quantum capacitance of CVD graphene upon hydrogenation. **a** Illustration of the field effect transistor setup fabricated from CVD graphene. **b** Room temperature conductance (*G*) plots as a function of the gate voltage (*V*_g_) showing the p-doping effect upon hydrogenation from 0 to 30 s. The gray dashed line is a guide-to-the-eye, highlighting the sublinear behavior of the *G*(*V*_g_) curves. **c** The shifts of the charge neutrality point (CNP) upon hydrogenation. **d** The carrier mobility of graphene, *µ*, vs the hydrogenation time. **e** Quantum capacitance *C*_q_ of graphene measured as a function of *V*_ch_ for 0–30 s of hydrogenation. **f** Impurity density *n*_imp_ vs hydrogenation time. The electrolyte solution is 0.1 M KCl with 10 mM Tris (pH 8). The error bars in **d**, **f** are the standard deviation of experimental values

For the device fabrication, we used high-quality, large-area CVD graphene following a facile and clean fabrication strategy as illustrated in Fig. 2a (see also Methods for details)^23^. Specifically, the topside of CVD graphene (on the copper foil) was first glued on the supporting glass substrate and protected by the photopolymer of pentaerythritol tetra(3-mercaptopropionate) and triallyl-1,3,5-triazine-2,4,6-trione (PETMP–TATATO)^24^. After the removal of backside graphene (by oxygen plasma), the copper ends were protected by covering them with a film of cellulose acetate butyrate (CAB). Then the graphene surface was exposed by etching the copper in a solution of ammonium persulfate, followed by a series of hydrogen plasma treatments to introduce defects with controlled densities. During the procedure, we employed a low-temperature annealing process (110 °C for ~1–3 h) to ensure a good adhesion of graphene on the underlying polymeric substrate. Only the fabricated graphene devices exhibiting mobilities on the order of 1000 – 1500 cm^2^ V^−1^ s^−1^ went through a series of field-effect, quantum capacitance, and cyclic voltammetry (CV) experiments immediately after each hydrogenation treatment. To rule out any possible sample-to-sample variations, all the aforementioned measurements were conducted on the same graphene samples.

Figure 2a depicts a GFET device with a source (S) and a drain (D) electrode bridged via a conductive graphene channel. A gate voltage (*V*_g_) is applied to the electrolyte solution via a Ag/AgCl reference electrode, to modulate the conductivity (*G*) of the GFET. Specifically, when the *V*_g_ is swept from negative to positive, the Fermi level (*E*_F_) of graphene shifts from the valence band (hole carriers) to the conduction band (electron carriers). At the so-called charge neutrality point (CNP), the concentration of hole carriers equals that of electron carriers and the conductivity of graphene reaches its minimum *G*_min_ (Fig. 2b). The slopes of the sublinear *G*(*V*_g_) curves are the measure for the carrier mobility *μ*, while the observed negative voltage of the CNP for untreated graphene implies an electron (n) doping induced by the underlying photopolymer substrate.

As hydrogenation proceeds, the CNP continuously shifts to more positive voltages, a characteristic of hole (p) doping (Fig. 2c). We attributed this doping effect to water adsorption, which occurs more readily on H-graphene than on untreated graphene^13,25^. Upon 1 s hydrogenation, graphene exhibits a slightly increased *G*_min_ (Fig. 2b) and a rather stable carrier mobility *μ* (Fig. 2d), suggesting that the mild hydrogenation treatment barely influences—even improves—the electrical properties of graphene^26^. As a result, we hypothesize that the H radicals after only 1 s of hydrogenation yields a cleaner graphene by effectively removing hydrocarbon adsorbates from the surface. Further hydrogenation reduces the mobility *μ* (and *G*_min_) of graphene down to ~750 cm^2^ V^−1^ s^−1^ (after 2 to 5 s of hydrogenation), after which *μ* stabilizes at 450–660 cm^2^ V^−1^ s^−1^ (after 5 to 30 s of hydrogenation). As a result, the introduction of only one H-*sp*^3^ defect per ~250,000 down to ~145,000* sp*^2^ hybridized carbon atoms effectively affects the mobility of charge carriers in graphene (correspondingly *L*_D_ = 45 nm down to 35 nm). In addition to the sublinear behavior of the *G*(*V*_g_) curves (even after a series of hydrogenation), the remarkable decrease of *G*_min_ also suggests that the conductivity of hydrogenated graphene is dominated by the so-called short-range scattering mechanism^27–29^. Such an observation is also confirmed by previous work in which hydrogenation introduced short-range scatterings in graphene lattice^30^.

### Quantum capacitance measurement

As a direct manifestation of the Pauli exclusion principle, the quantum capacitance effect in graphene is particularly prominent due to its low DOS^31^. The quantum capacitance *C*_q_ of graphene, can be directly determined as a function of the channel potential across the graphene sheet *V*_ch_ using an electrochemical configuration (Supplementary Figure 4)^32^. In Fig. 2e, the measured *C*_q_ generally displays a broad minimum, *C*_q,min_, at the voltage of the CNP and linearly increases with *V*_ch_ on both sides of the CNP. Similarly to the changes in conductivity after hydrogenation (Fig. 2b), the V-shaped *C*_q_(*V*_ch_) curves exhibit not only positive CNP shifts, but also broader and decreased minimums with increasing hydrogenation times. In nature, *C*_q,min_ is directly related to the density of effective charged impurities n^*^ (since these impurities can cause local potential fluctuations in graphene), which can reveal the global behavior of defects in graphene (Supplementary Note 2)^19,33^. Notably, the capacitance we measured (as well as n^*^ values) are generally lower than those reported in previous studies. For example, untreated graphene presents a n^*^ = 9.73 × 10^10^ cm^−2^, ~8 times lower than CVD graphene on Si/SiO_2_ wafer (n^*^ = 8.0 × 10^11^ cm^−2^)^32^. Such a remarkably lowered n^*^ can be ascribed to our clean fabrication strategy (Methods) which introduces less charged impurities, or reflects the differences between the substrates, which could lead to different degrees of charge transfer.

More importantly, the effective charged impurities n^*^ is proportional to the impurity density *n*_imp_, referred as the impurities at the interfaces between graphene and the substrate, or between graphene and air, or resulting from the intrinsic defects caused by the growth or transfer of CVD graphene. In Fig. 2f, *n*_imp_ decreases in the first 5 s and then settles till 30 s hydrogenation, a scenario suggesting that the mild hydrogenation (within 1–5 s) sweeps away the trapped/adsorbed charge impurities at graphene interfaces. The evolution of the defect density *n*_D_ and of the impurity density *n*_imp_ are closely related and critical to the electron transport and electrochemical kinetics, which we will discuss in more detail below (section Correlation of *n*_D_ with *n*_imp_).

### Electrochemical kinetics measurement

We employed cyclic voltammetry (CV) to investigate the electrochemical behavior of H-graphene. Specifically, we used the hexaammineruthenium (II)/hexaammineruthenium (III) redox couple (Sigma Aldrich), Ru(NH_3_)_6_^2+/3+^, as an outer-sphere redox mediator: (i) it is surface insensitive and thus the electron transfer from the mediator to graphene (and vice versa) mainly relies on the electronic structures of the electrode and of the mediator itself and (ii) it possesses a standard potential in the vicinity of the Fermi level of graphene^34^.

**Fig. 3:**
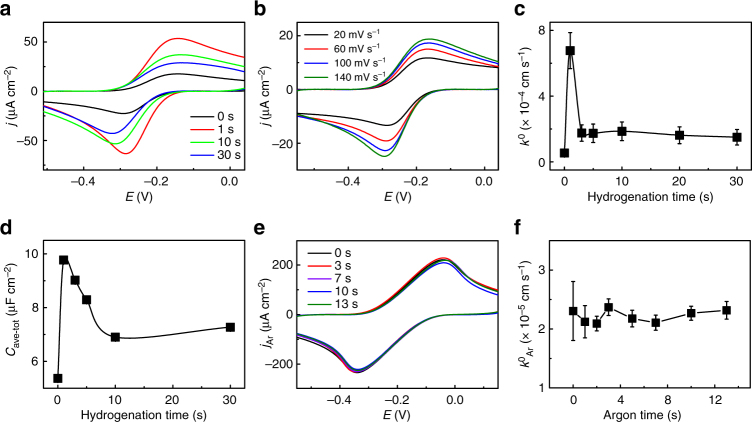
Electrochemical behavior of CVD graphene upon hydrogenation. **a** Cyclic voltammograms (CVs) obtained on graphene after 0–30 s of hydrogenation at a scan rate of 100 mV s^−1^. **b** Current density vs scan rate for untreated graphene shown in **a**. **c ** The electron transfer rate *k*^0^ vs hydrogenation time from 0 to 30 s. **d** The averaged total capacitance *C*_ave−_ _tot_ vs hydrogenation time from 0 to 30 s. **e** CV curves obtained on graphene after 0–13 s of Ar treatment at a scan rate of 100 mV s^−1^. **f** $k^0_{{\mathrm{Ar}}}$ vs argon plasma treating time from 0 to 13 s. The aqueous electrolyte solution contains 0.1 M KCl supplemented with 10 mM Tris at pH 8. The redox probe employed is 1 mM hexaammineruthenium (II)/hexaammineruthenium (III) chloride. The error bars in **c**,** d**, **f** are the standard deviation of experimental values

From the CVs in Fig. 3a, we determined the electrochemical activity of graphene towards the redox probe before and after 1–30 s of hydrogenation. The current densities (*j*) of the oxidation peak (at –170 mV) and reduction peak (at –290 mV) show that 1 s of hydrogenation is sufficient to increase the electrochemical activity of graphene by a factor of four compared to untreated graphene, while further hydrogenation brings about an immediate decrease of activity. The peak-to-peak separation (Δ*E*_p_), a qualitative indicator of the electrochemical reversibility in graphene, displays a minimum at 1 s of hydrogenation, which is in agreement with the observed maximum for the current density (Supplementary Figure 5c).

Furthermore, from the data in Fig. 3b we extracted the heterogeneous electron transfer rate (*k*^0^) between the graphene basal plane and the redox probe to quantitatively evaluate the electrochemical kinetics of graphene upon hydrogenation. Specifically, Δ*E*_p_ of the quasi-reversible redox peaks are below 220 mV as the scan rate (*v*) increases, which meets the criteria of Nicholson’s method to estimate the kinetic parameters^34,35^ (Supplementary Note 3). Consistent with the current density depicted in Fig. 3a, the deduced values of *k*^0^ exhibit a peaked behavior as a function of the hydrogenation time (Fig. 3c). In details, *k*^0^ increases by up to ~12 fold (6.77 × 10^−4^ cm s^−1^) after 1 s of hydrogenation compared to untreated graphene (5.37 × 10^−4^ cm s^−1^). For longer hydrogenation times, *k*^0^ sharply drops down to ~1.70 × 10^−4^ cm s^−1^ within 5 s and stabilizes at 1.50 × 10^−4^ cm s^−1^ after 30 s hydrogenation. Such a trend is reproducible for different batches (Supplementary Figure 5a, b and Supplementary Note 4).

The total electrical capacitance (*C*_tot_) per unit area of graphene, consists of the electrical double layer capacitance (*C*_dl_) and the graphene quantum capacitance (*C*_q_) connected in series^36^. *C*_tot_ can be obtained either by using a lock-in technique (Methods) or by measuring the capacitive CV current for different scan rates, which is an averaged evaluation over a relatively wide potential (*C*_ave−tot_, Supplementary Figure 6 and Supplementary Note 5). Additionally, the basic rectangular shapes of the capacitive current curves imply a purely capacitive behavior without Faradaic processes. Furthermore, upon hydrogenation *C*_ave−tot_ first increases after 1 s, then drops till 10 s and saturates till 30 s, varying similarly as *k*^0^ (Fig. 3d). The observed changes in *C*_ave−tot_ can be mainly attributed to the DOS variations with hydrogenation (as *C*_q_ dominates in the series circuit).

### Electrochemistry of H-*sp*^3^ vs vacancy defects

To further evaluate the exact impact of defects on the electrochemical kinetics of graphene, we also studied samples that were treated with an argon plasma (referred as Ar-graphene) with comparable defect densities as to hydrogenated graphene (Supplementary Figure 7 and Supplementary Note 6). In contrast to the sensitive electrochemical behavior in H-graphene (Fig. 3a, c), both the current density and *k*^0^ on Ar-graphene show negligible sensitivity to the argon plasma treatment (Fig. 3e, f). Such trends are consistent with the previous report that a low density of vacancy defects hardly impacts the electrochemical activity of graphene^26^.

Based on the different *I*(D)/*I*(D′) ratios characterized using Raman spectroscopy (i.e., ~7 for Ar-graphene and ~10 for H-graphene)^37^, we identify that argon plasma forms vacancy defects by removing carbon atoms, while hydrogenation changes graphene hybridization from *sp*^2^ to *sp*^3^. Thus, we gain insight into the driving mechanism for the observed electrochemical behavior. Rather than the vacancy defect, the change of hybridization (in H-graphene) is closely related to the electrochemical properties of hydrogenated graphene (Fig. 3). Meanwhile, coincident to the boost of *k*^0^, the *G*_min_ and *μ* increase slightly after 1 s of hydrogenation (Fig. 2), indicating a cleaner graphene with less surface scattering centers: hydrogen radicals are expected to react with the hydrocarbons adsorbed on the surface of graphene. Such a cleaning effect is due to the much higher reactivity of hydrocarbons with hydrogen radicals compared to the reactivity of the graphene itself with the same radicals. As airborne contaminations, hydrocarbons can adsorb onto any surface, as revealed from the observation that the wetting of graphitic surface dramatically changes over short time periods^38^. Indeed, such cleaning effect is in agreement with prior observations that graphite—more specifically freshly exfoliated highly oriented pyrolytic graphite—exhibits high but instantly decaying electrochemical activity due to the exposure to airborne contaminants^39,40^. Notably, we expect no cleaning effect using argon plasma under our condition (ion energy ~60 eV)^41^, as also confirmed by the high-resolution transmission electron microscopy images of Ar-graphene (not shown here).

In addition, we employed X-ray photoelectron spectroscopy (XPS, Supplementary Figure 8 and Supplementary Note 7) in complementary to Raman to compare graphene containing similar defect densities (according to Raman analysis) after 60 s of hydrogenation and after 15 s of argon plasma treatment. The presence of C-*sp*^2^ (284 eV), C-*sp*^3^ (285 eV), C–O (286–286.2 eV), and C = O (287.8–288 eV) in C 1*s* spectra, suggest that both samples contain *sp*^2^ and *sp*^3^ carbon with minor oxygen contaminants from PMMA residues (only used for XPS samples to transfer graphene onto the Si substrate). As XPS probes both the surface chemistry of graphene and its surface adsorbents, we observed a higher content of *sp*^3^ carbon from XPS analysis (Supplementary Table 1), compared to the results of Raman spectroscopy. Thus, we ascribe the observed *sp*^3^ C in both H-graphene (6.2–8.0%) and Ar-graphene (3%) to possible surface adsorbents including PMMA residues and hydrocarbons. A trace amount of *sp*^3^ doping in the graphene lattice (up to 0.8% *sp*^3^ C in H-graphene) was, however, determined by Raman spectroscopy.

### Correlation of *n*_D_ with *n*_imp_

To shed light on the influence of the electrochemical current on the performance of GFET sensors, we discuss here the interplay between the in-plane charge transport and the electrochemical activity of H-graphene. Particularly, we systematically investigate the correlations between the DOS, the mobility of charge carriers *μ*, and the electron transfer rate *k*^0^, with respect to the density of charged impurity *n*_imp_ and defect density *n*_D_. Finally, we provide a comprehensive discussion on the driving mechanism for the electrical and electrochemical behavior we observe for H-graphene.

**Fig. 4:**
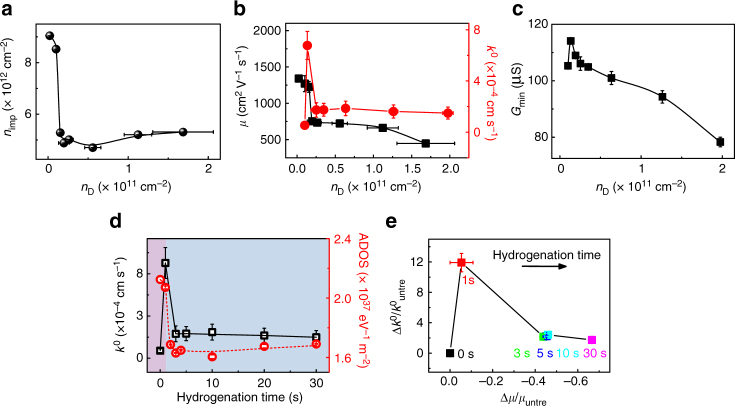
Quantum and electrochemical interplays in hydrogenated graphene. **a** The dependence of *n*_imp_ on *n*_D_. **b** Correlations of *μ* and *k*^0^ with *n*_D_, respectively. **c** The minimum conductivity (*G*_min_) vs *n*_D_. **d** The correspondence between ADOS and *k*^0^ as a function of the hydrogenation time. The purple region represents the cleaning-dominated regime and the blue region represents the regime where the chemical modification dominates. **e** The relative variations of Δ*μ*/*μ*_untre_ correlating with $\Delta k^0/k_{{\mathrm{untre}}}^0$ according to the corresponding hydrogenation time. The subscript "untre" represents the untreated graphene. The error bars are the standard deviation of experimental values

To understand the correlation between defect density *n*_D_ and impurity density *n*_imp_ (presented in Fig. 4a), it is important to consider their relation to the electronic properties of graphene. It is well-known from studies on supported graphene that defects yield short-range electron scattering in graphene. Impurities, on the other hand, cause long-range (Coulomb) scattering resulting in trapped electron states. The overall conductivity of graphene is dictated by the prevalence of either impurities or defects in the sample; *n*_D_ dominates at high charge carrier density while *n*_imp_ determines at low charge carrier density^28,42^. Impurities are generally present at the interfaces between graphene and air or between graphene and the underlying substrate. The cleaning effect in the first second of hydrogenation appears in Fig. 4a as the decreasing onset for *n*_imp_ (from 9.05 to 8.52 × 10^12^ cm^−2^).

Aside from the cleaning effect, the subsequent dramatic drop in *n*_imp_ from 8.52 × 10^12^ cm^−2^ to 5.01 × 10^12^ cm^−2^ (2–5 s) can be ascribed to the hybridization change from *sp*^2^ to *sp*^3^ when *n*_D_ steadily increases with hydrogenation. The presence of *sp*^3^ hybridized spots in the lattice causes the lattice to expand and to curve. The increased distance between the lattice and substrate-related impurities explains the sharp drop in *n*_imp_. Further on, *n*_imp_ slightly increases (from 5.01 × 10^12^ cm^−2^ to 5.31 × 10^12^ cm^−2^), which can be ascribed to the accumulation of trapped water molecules at the graphene surface, accompanying the increasing *n*_D_ (*n*_D_ > (2.6 ± 0.5) × 10^10^ cm^−2^).

### Correlation of *k*^0^ and *μ* with *n*_D_

The first report on the correlation of *k*^0^ with the density of vacancy defects in monolayer graphene showed that *k*^0^ remained constant at low densities but underwent a tenfold increase at a defect density of 10^12^ cm^−2^ (*I*(D)/*I*(G) ≅ 2.95)^26^. However, the high density of vacancy defects lowers the electrical performance of graphene. In our work, *k*^0^ is improved up to 12-fold (to 6.77 × 10^−4^ cm s^−1^) at a low H-*sp*^3^ defect density of *n*_D_ = (1.0 ± 0.1) × 10^10^ cm^−2^ (*I*(D)/*I*(G) ≅ 0.4). Then, when *n*_D_ continues to rise, *k*^0^ drops sharply to stabilize between 1.5 and 1.7 × 10^−4^ cm s^−1^ (red line, Fig. 4b).

Separately, when *k*^0^ increases, the carrier mobility *μ* stays unchanged (or becomes slightly higher) compared to untreated graphene (black line, Fig. 4b) at *n*_D_ = (1.0 ± 0.1) × 10^10^ cm^−2^. With the continuous growth of the defect density up to *n*_D_ = (2.0 ± 0.7) × 10^10^ cm^−2^, *μ* exhibits a deep drop, indicating that the carrier transport in graphene is sensitive to the existence of even low densities of H-*sp*^3^ defects (*n*_D_ ≤ (2.0 ± 0.7) × 10^10^ cm^−2^ corresponding to a distance *L*_D_ of ~40 nm between the defects). For higher defect densities, however, the decrease of *μ* is less pronounced (till *n*_D_ = (1.7 ± 0.4) × 10^11^ cm^−2^, that is *L*_D_~14 nm). The minimum conductivity (*G*_min_) changes with *n*_D_ in Fig. 4c, correlating well with the fluctuations in mobility (Fig. 4b).

Based on the Boltzmann theory, the conductivity of graphene (*G*) is proportional to 1/(*n*_D_)^1/2^ at high carrier density (far from the CNP)^43^. In consequence, *μ* is expected to decrease with increasing *n*_D_ upon hydrogenation. Meanwhile, at low carrier density (near the CNP), *G* is proportional to (*n*_imp_)^1/2^ and *G*_min_ is expected to reduce with the decrease of *n*_imp_. The data in Fig. 4b, c fit the theory, except for the increase in both *G*_min_ and *μ* upon the initial hydrogenation (*n*_D_ = (1.0 ± 0.1) × 10^10^ cm^−2^). This can be explained by considering the cleaning of adsorbates from the graphene surface. Particularly, hydrogenation slightly introduces H-*sp*^3^ defect while also removing surface short-range scatters outweighing the effect on the conductivity and mobility of graphene. Separately, the decrease of the DOS after hydrogenation contributes to the decrease of the density of intrinsic charge carrier *n* instead of affecting the carrier mobility of these charge carriers in graphene.

### Correlation between the DOS and *k*^0^

In electrochemistry, the kinetics of electron transfer from graphene to a redox probe is dependent on the electrochemical potential of electrons in graphene (that is the Fermi level, *E*_F_) with respect to the electrochemical potential of the redox couple in solution^31,44^. For example, for the electron to flow from the redox probe to graphene, the graphene *E*_F_ that can be tuned by varying the potential applied to the graphene electrode or by sweeping the gate voltage, should at least align with the LUMO level of the oxidative molecule to allow an efficient electron transfer. For a non-adiabatic process, the DOS in graphene decides—whether or not—a basal plane electron could tunnel to the redox probe. Practically, the electron transfer occurs when the electronic resonance between the redox molecule and graphene is reached, that is for a given value of the applied potential, and is measured by studying how fast the electron transfer reaction can reach its equilibrium (kinetics)^45^. In short, the electrochemical kinetics (reflected by *k*^0^) of graphene relies on the DOS on the premise of non-adiabatic electron transfer.

In 2D materials like graphene, its minimal quantum capacitance,* C*_q,min_, can be used to deduce its average DOS (ADOS) at a specific *E*_F_: *ρ* = *C*_q,min_/*e*^2^, where *e* is the electron charge^46^. In Fig. 4d, we therefore plot and compare the ADOS with *k*^0^ as hydrogenation proceeds. During the first second (within the purple region), the ADOS decreases a little, however *k*^0^ increases dramatically, which can be mainly ascribed to the volatilization of hydrocarbon contaminants. That is, the hydrogen radicals first remove the hydrocarbon adsorbates to reveal the electrochemical activity of the underlying graphene, as the kinetic process involves interface-sensitive electron tunneling. Notably, H radicals can also attack the graphene lattice during the hydrocarbon cleaning and the resulting H-*sp*^3^ defect could lead to the observed decrease in the ADOS^26^. Upon further hydrogenation (the beginning of the blue region), the ADOS and *k*^0^ decrease sharply, which are mainly due to the modification of the graphene basal plane by hydrogen radicals. The decay of *k*^0^ with DOS agrees with the non-adiabatic electron transfer, in which the rate depends on the electronic properties of the electrode due to the weak electronic interaction between the redox mediator and the electrode, according to the Levich–Dogonadze theory^47^ and Fermi’s golden rule^48^. We would like to note here that the decrease in *k*^0^ (Fig. 4d) is unlikely due to H-*sp*^3^ termination, as the formed C–H dipole is more susceptible towards nucleophilic attack^49^, which could increase the electrochemical activity. Nor is it likely that the kinetics were affected by surface oxidation during exposure to air: XPS spectra demonstrate the negligible oxidation of H-graphene after its exposure to the ambient conditions even for 1 week (Supplementary Figure 8 and Supplementary Table 1). Additionally, the DOS was predicted to contribute more significantly to the kinetics compared to surface modification^40^. Thus we demonstrate for the first time, that the electrochemical kinetics in the single layer graphene is highly sensitive to the ADOS upon the addition of even a single H-*sp*^3^ defect per 100,000 *sp*^2^ carbon atoms. More importantly, the correlation between *k*^0^ and DOS in return confirms the importance of graphene electronic properties (DOS) in terms of defining the electrochemical current for sensing application.

### Correlation between *μ* and *k*^0^

Figure 4e shows the dependence of the relative variation of $\Delta k^0/k_{{\mathrm{untre}}}^0$ with Δ *μ*/*μ*_untre_, where $\Delta k^0/k_{{\mathrm{untre}}}^0 = \frac{{k^0 - k_{{\mathrm{untre}}}^0}}{{k_{{\mathrm{untre}}}^0}}$, $\Delta \mu /\mu _{{\mathrm{untre}}} = \frac{{\mu - \mu _{{\mathrm{untre}}}}}{{\mu _{{\mathrm{untre}}}}}$, and the subscript “untre” denotes untreated graphene. Notably, the negative values of $\Delta \mu /\mu _{{\mathrm{untre}}}$ corresponds to the degradation of the carrier mobility upon hydrogenation time (see also Fig. 2). Specifically, the peak value of the $\Delta k^0/k_{{\mathrm{untre}}}^0$ after 1 s of hydrogenation is ascribed to the disclosure of the intrinsic electrochemical activity of the graphene basal plane resulting from the volatilization of hydrocarbon adsorbates. For hydrogenation times longer than 3–5 s, $\Delta k^0/k_{{\mathrm{untre}}}^0$ decreases by ~5 times compared to the peak value (at 1 s) with preserved mobility (~50–60%). Our results therefore suggest the importance of H-*sp*^3^ defects towards achieving a low electrochemical activity in GFET by suppressing its DOS. Interestingly, the boosted *k*^0^ upon H_2_ plasma cleaning reveals a relatively high electrochemical activity of the graphene basal plane, which was often believed to be inert and inactive in electrochemistry^50^.

## Discussion

We demonstrated that a hydrogen radical plasma cleans the surface of graphene and chemically modifies the graphene lattice upon continuous exposure. In the beginning (the first 1–5 s), the introduced H radicals mainly sweep the hydrocarbon adsorbates away from the graphene surface. In particular, within the first second of hydrogenation we observed a large enhancement of the electrochemical activity on the surface of pristine graphene (with a minimum of H-*sp*^3^ defects). We postulate that in untreated graphene, the electrochemical activity was  initially blocked by the presence of hydrocarbon adsorbates which are now removed by the hydrogen plasma^51^ (Fig. 3c and Fig. 4d). Remarkably, even traces amounts of H-*sp*^3^ defects in graphene (only one *sp*^3^ defect per ~400,000 carbon atoms) results in the decrease of the DOS, a quantity considerably sensitive to the changes of electronic and chemical properties of graphene. Additionally, further hydrogenation of the graphene basal plane largely depresses *k*^0^ down to one fifth of its original value (pristine graphene), presumably by lowering its DOS. Interestingly, however, the mobility of graphene is preserved to a large extent (Fig. 2), promising future development of electrochemical field-effect transistors based on H-graphene.

Besides hydrogenation, the physisorption of water molecules at the graphene surface reflected by the observed p-doping effect (Fig. 2b)^25^ is also considered. As non-covalent functionalization, water molecules can barely disturb the intrinsic aromaticity^52^, thus we expect that it exerts little impact on the electronic structure and electrochemistry of graphene^53^. For example, the resistivity at the CNP as well as the carrier mobility barely changed after removal of the adsorbed water^25^. Separately, negligible oxidation is found using XPS characterization even in aged graphene, as shown in Supplementary Figure 5. Therefore we can exclude the major contributions of surface-adsorbed water and graphene oxidation to the observed electrical and electrochemical properties of hydrogenated graphene.

In summary, we have systematically probed the interplay between the in-plane electron transport and the electrochemical activity of the graphene basal plane by modulating the density of H-*sp*^3^ defects. Interestingly, the mild hydrogenation within 1–5 s largely preserves the basic electrical mobility while effectively depresses the electrochemical kinetics *k*^0^ and lowers the DOS in graphene, manifesting as a plausible way to improve the sensitivity of GFET. For the first time, we demonstrated that the electrochemical kinetics in single layer H-graphene is highly dependent on the ADOS, which supports the theory of non-adiabatic electron transfer on graphene. Additionally, the electrochemical activity of the pristine graphene basal plane can be restored by the removal of surface-adsorbed hydrocarbons using a low dose of hydrogen radicals, a result that will further promote graphene as an electrode for electrochemical studies. The correlation between the carrier mobility and the electrochemical kinetics suggests that the electrical conductivity of H-graphene is an important parameter to consider, for example, in GEC sensors. We believe our work will inspire several research communities to consider hydrogenated graphene as a potent material for sensing applications with performances going beyond previously reported (G)FET sensors.

### GFET device fabrication

To fabricate the GFET devices, the graphene side of the copper growth substrate (CVD graphene, Graphenea S.A.) is glued to a glass slide with a PETMP–TATATO polymer^23^. PETMP–TATATO (Sigma Aldrich) is a clean and biocompatible polymer usually used for dental restorative application^24^. After sufficient photo-initiated crosslinking reaction at room temperature (12 h in daylight), the whole stack (glass-glue-graphene-copper) was oxidized with an O_2_ plasma (60 W/0.5 mbar/2 min) to remove the trace of graphene that had grown on the backside of the copper substrate (i.e. the side now facing to the air). To fabricate the source and drain electrodes, both ends of the copper substrate (a strip of copper) were protected by a polymer film of cellulose acetate butyrate (CAB, 30 mg mL^-1^ in ethyl acetate, Sigma Aldrich). Then an ammonium persulfate solution (0.5 M) was used to etch the non-protected copper foil to reveal the clean CVD graphene supported by the photopolymer and glass substrate without any possible polymer residues. Finally, the fabricated graphene devices were exposed to a hydrogen plasma for different durations to introduce defects with controlled densities.

### Thiol–enes polymer

Commercially available pentaerythritol tetra(3-mercaptopropionate) and triallyl-1,3,5-triazine-2,4,6-trione (referred to as PETMP and TATATO, respectively) are used as monomers for the thiol-ene resin formulation. 4:3 volume proportion of PETMP–TATATO were selected for the preparation of the photopolymer.

### Plasma condition

Capacitively coupled plasma system with the radio-frequency (RF) of 40 kHz and 200 W power from Diener electronic (Femto) was employed at room temperature. The base pressure of this system is <0.02 mbar. The parameters used for the controlled introduction of defects were 10 W/1.0 mbar for hydrogen plasma and 8 W/0.85 mbar for argon plasma. Specifically, a Faraday cage with grid was employed to shield all the energetic hydrogen ions to form a mild radical plasma to react with graphene.

### Characterization

Raman spectroscopy and mapping were collected from both exfoliated graphene and CVD graphene (using the PMMA transfer method^54^) on Si/SiO_2_ substrate. Raman spectra of CVD graphene on PETMP-TATATO polymer was also performed (Supplementary Figure 2a). The Raman spectrometer used is a WITEC alpha300 R-Confocal Raman Imaging with a laser wavelength of 532 nm. To minimize the potential damage from laser heating effect, the laser power was controlled under 1.1 mW. All of the measurements were performed under ambient conditions at room temperature. XPS data were collected from a K-Alpha X-ray photoelectron spectrometer by Thermo Scientific. SEM images were carried out on a JEOL SEM 6400 microscope. A JPK NanoWizard Ultra Speed AFM was employed to characterize the topology of exfoliated graphene before and after hydrogenation on a Si/SiO_2_ substrate. The images were scanned in an intermittent contact mode in air at room temperature.

### Electrical measurement

The transport measurements of GFET devices upon different hydrogenation times were performed on a SR830 DSP lock-in amplifier with narrow filters. Electrolyte- or electrochemical-gated GFET measurements were carried out in 0.1 M KCl solution containing 10 mM Tris as the buffer (pH 8, both from Sigma Aldrich). The gate voltage was applied on a AgCl/Ag wire as the reference electrode, at a sweep rate at 100 mV s^−1^, while the source/drain current was fixed at 0.1 µA.

### Quantum capacitance

As illustrated in Supplementary Figure 4, the total capacitance *C*_tot_ of an electrolyte-gated GFET, is composed of two components in series, quantum capacitance *C*_q_ and the electric double-layer capacitance *C*_dl_. The *C*_dl_ for the KCl solution can be approximated as 10–20 µF cm^−2^ for a wide range of ionic concentration >1 mM^55^. *C*_q_ is relatively small (~1 µF cm^−2^) compared to the *C*_dl _(in series) and thus dominates the total capacitance *C*_q_ ~ *C*_tot_^32^. By calculating the *C*_q_ based on 1/*C*_tot_ = 1/*C*_q_ + 1/*C*_dl_, we get the curves of *C*_q_ vs the potential distributed on graphene channel *V*_ch_ (*V*_ch_ = (*V*_g_*C*_dl_)/(*C*_dl_ + *C*_q_)) for different hydrogenation times.

### Electrochemical measurement

The electrochemical experiments were carried out in a homemade one-compartment three-electrode electrochemical cell at ambient conditions. The working electrode is the CVD grown graphene and the counter electrode a platinum wire. All potentials in this work are reported with respect to a saturated Ag/AgCl reference electrode. A potentiostat/galvanostat (CompactStat, Ivium Technologies) was used for the electrochemical measurements. The electrolyte, 0.1 M KCl, was prepared from KCl (Sigma Aldrich, ≥98%) and ultrapure water (Millipore Milli-Q gradient A10 system, 18.2 MΩ cm). The measured current was normalized to the geometric surface area of the working electrode and not corrected for Ohmic drop as the obtained currents were very low. Prior to the experiments, the cell containing the electrolyte solution was purged with argon to remove the dissolved oxygen.

### Data availability

The data that support the findings of this study are available from the corresponding author on request.

## Electronic supplementary material

Supplementary Information
