# Supplementary material for: Quantum and electrochemical interplays in hydrogenated graphene
Source: Nat Commun. 2018 Feb 23;9:793. doi: 10.1038/s41467-018-03026-0 (PMC5824792; doi:10.1038/s41467-018-03026-0)
Supplement: Supplementary file 1 — Supplementary Information [file 41467_2018_3026_MOESM1_ESM.pdf]

Supplementary Information

Quantum and electrochemical interplays in hydrogenated graphene

Jiang et al.

Supplementary Figures

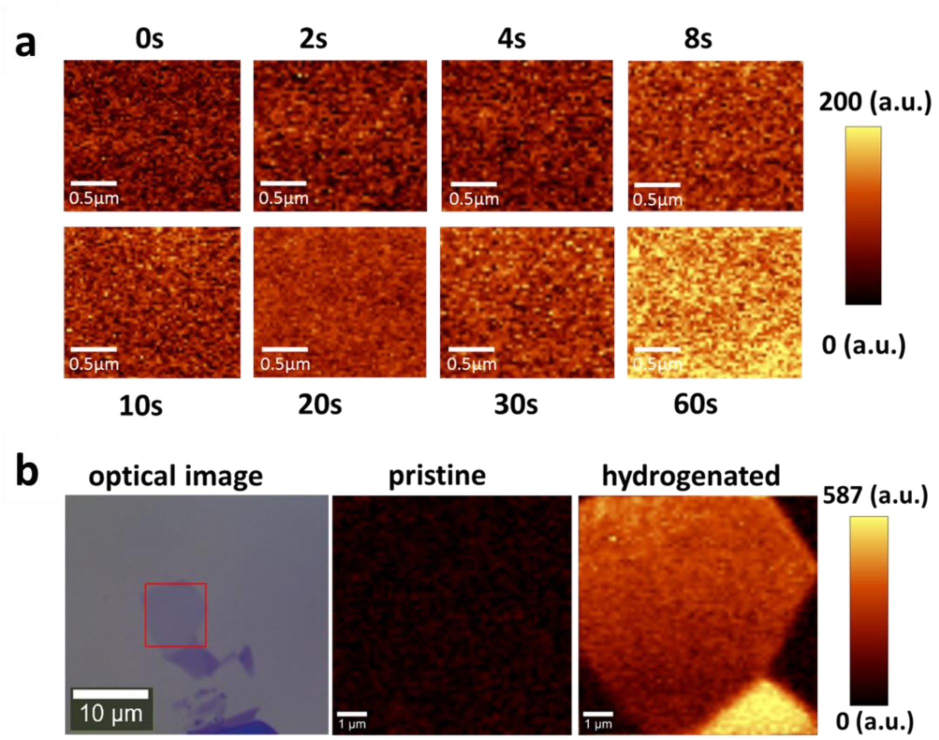

Supplementary Figure 1 │ Raman mapping of graphene upon hydrogenation. a, Maps of the D band intensity of CVD graphene with hydrogenation time ranging from 0 to 60 s. **b,** Maps of the D band intensity for pristine (0 s) and hydrogenated graphene (60 s). Graphene was obtained by exfoliation of natural graphite.

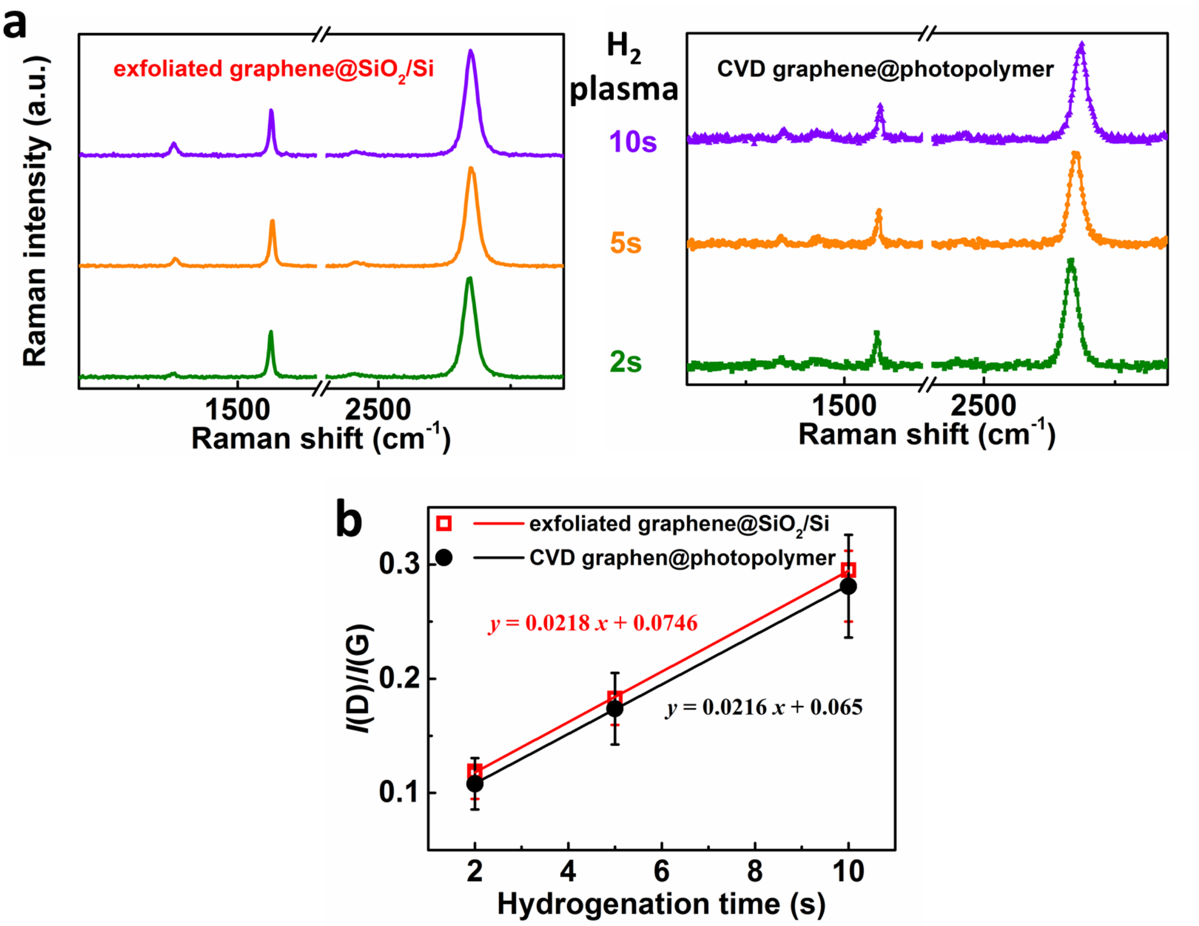

Supplementary Figure 2 │ Raman spectra on exfoliated and CVD graphene. a, Raman spectra collected from exfoliated (left panel) and CVD (right panel) graphene after 2 s, 5 s and 10 s of hydrogenation. **b,** The *I*(D)/*I*(G) ratio extracted from exfoliated (red hollow square) and CVD (black solid dot) graphene. The error bars in **b** are defined by the standard deviation of experimental values

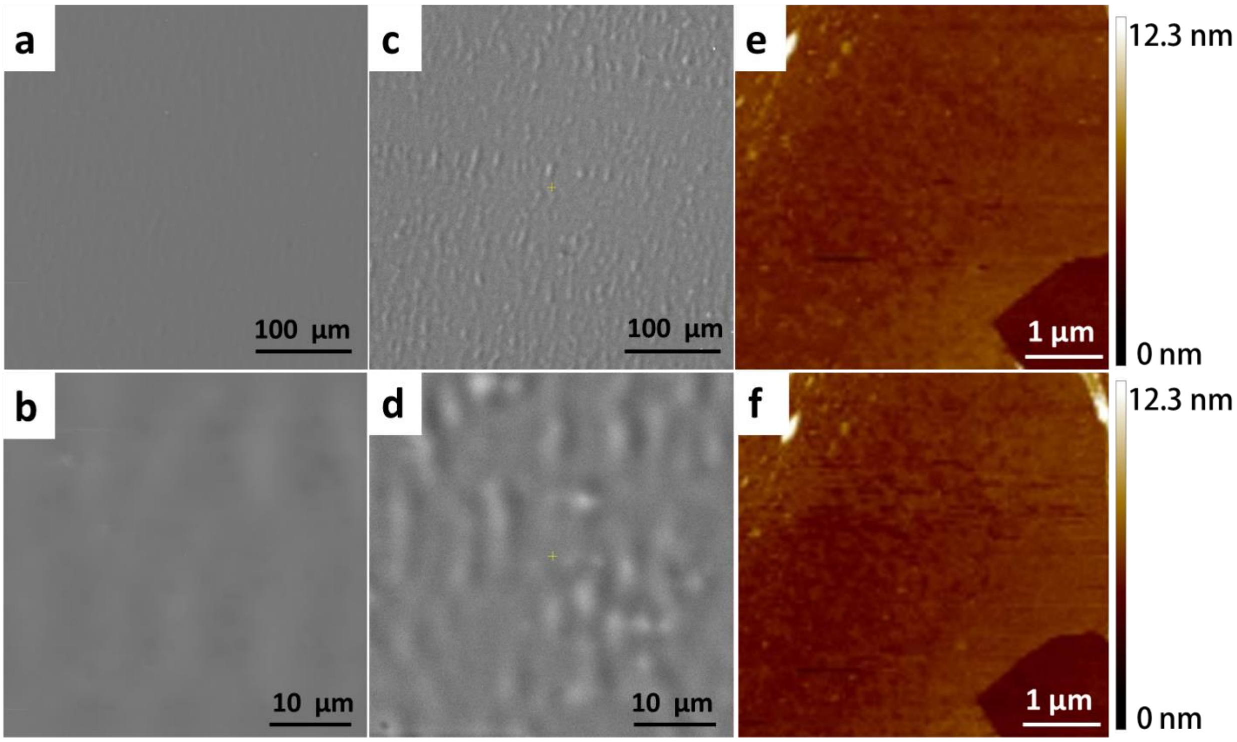

Supplementary Figure 3 | Scanning electron microscopy (SEM) and atomic force microscopy (AFM) of hydrogenated graphene. SEM of hydrogenated graphene on a PETMP-TATATO polymer before (a, b) and after (c, d) hydrogenation. AFM of exfoliated graphene on Si/SiO_2 before (e) and after (f) hydrogenation.

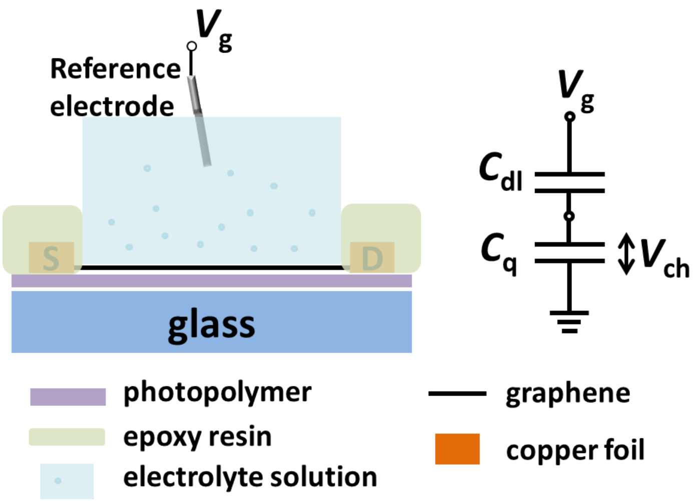

Supplementary Figure 4 │ Quantum capacitance measurement setup and circuit illustration.

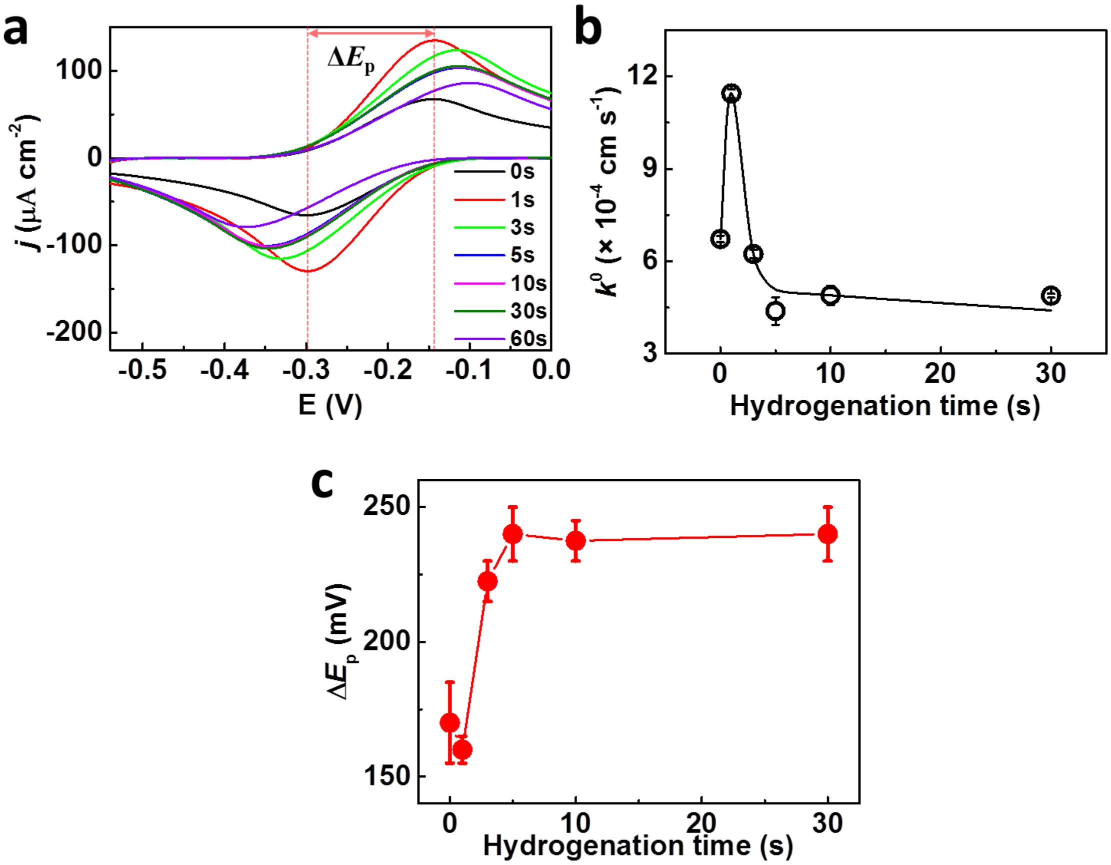

Supplementary Figure 5 | Cyclic voltammograms of hydrogenated graphene. a, Cyclic voltammograms (CVs) collected from the same graphene sample after sequential hydrogenation treatments. The electrolyte solution is 0.1 M KCl containing 10 mM Tris and 1 mM $Ru(NH_3)_6Cl_2$ /$Ru(NH_3)_6Cl_3$, respectively. **b**, The evolution of electron transfer rate k^0, along hydrogenation time, extracted from the CVs plotted in a). **c**, The peak to peak separation (ΔE_p) vs the hydrogenation time. The error bars in **b**,**c** are defined by the standard deviation of experimental values

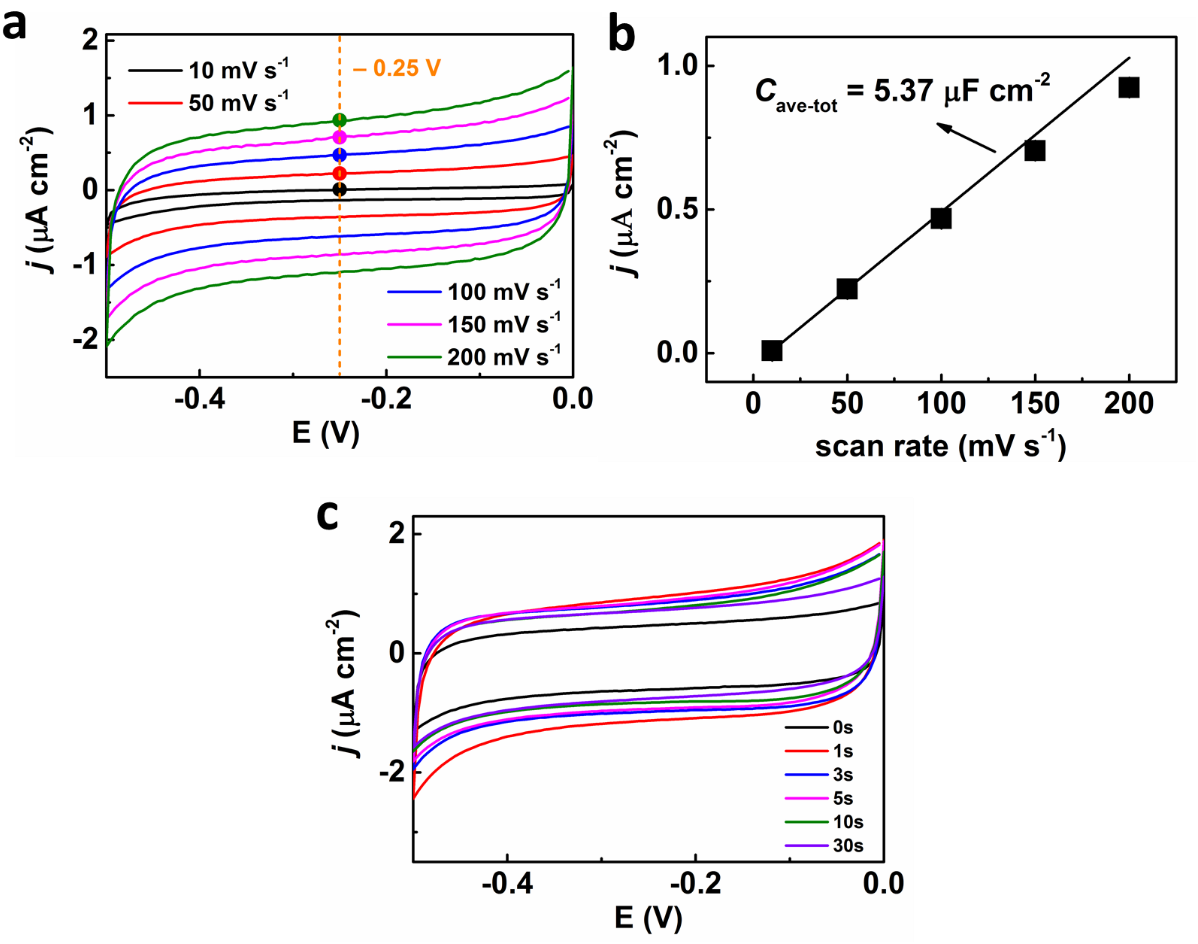

Supplementary Figure 6 │ Capacitance measurement for hydrogenated graphene. **a**, Cyclic voltammograms (CVs) of untreated graphene in 0.1M KCl solution (containing 10 mM Tris) as a function of the scan rate ranging from 10 to 200 mV s^{-1}. **b**, The plot of the current density (positive-going scan) versus scan rate for an applied potential of –0.25 V. **c**, Capacitive CVs obtained on graphene before and after 1 to 30 s of hydrogenation. The error bars in **b** are defined by the standard deviation of experimental values

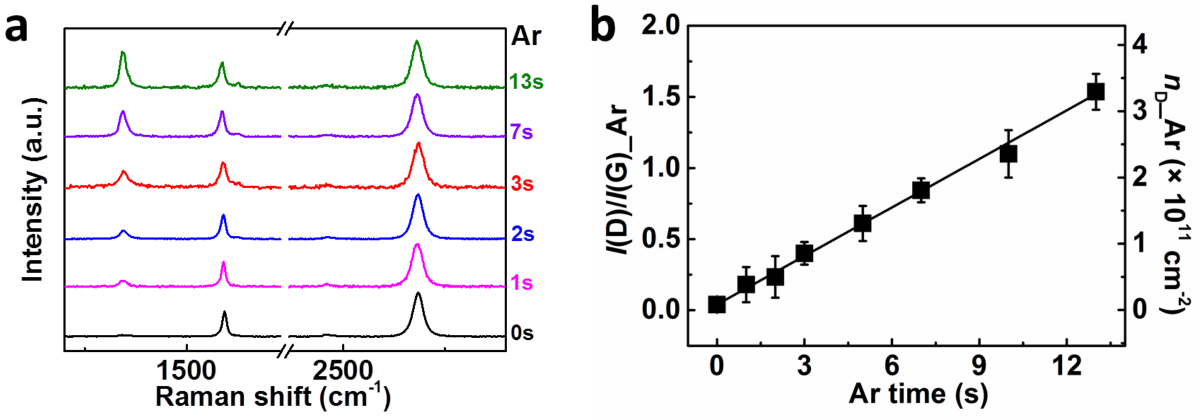

Supplementary Figure 7 │ Raman spectroscopy and electrochemistry of Ar-graphene. a, Averaged Raman spectra of CVD graphene transferred on a Si/SiO_2 substrate after the Ar ion plasma (8 W, 0.85 mbar) for 0-13 s. **b**, The intensity ratio $I(D)/I(G)$_Ar and the derived defect density n_D_Ar, plotted vs. the Ar bombarding time. The error bars in **b** are defined by the standard deviation of experimental values

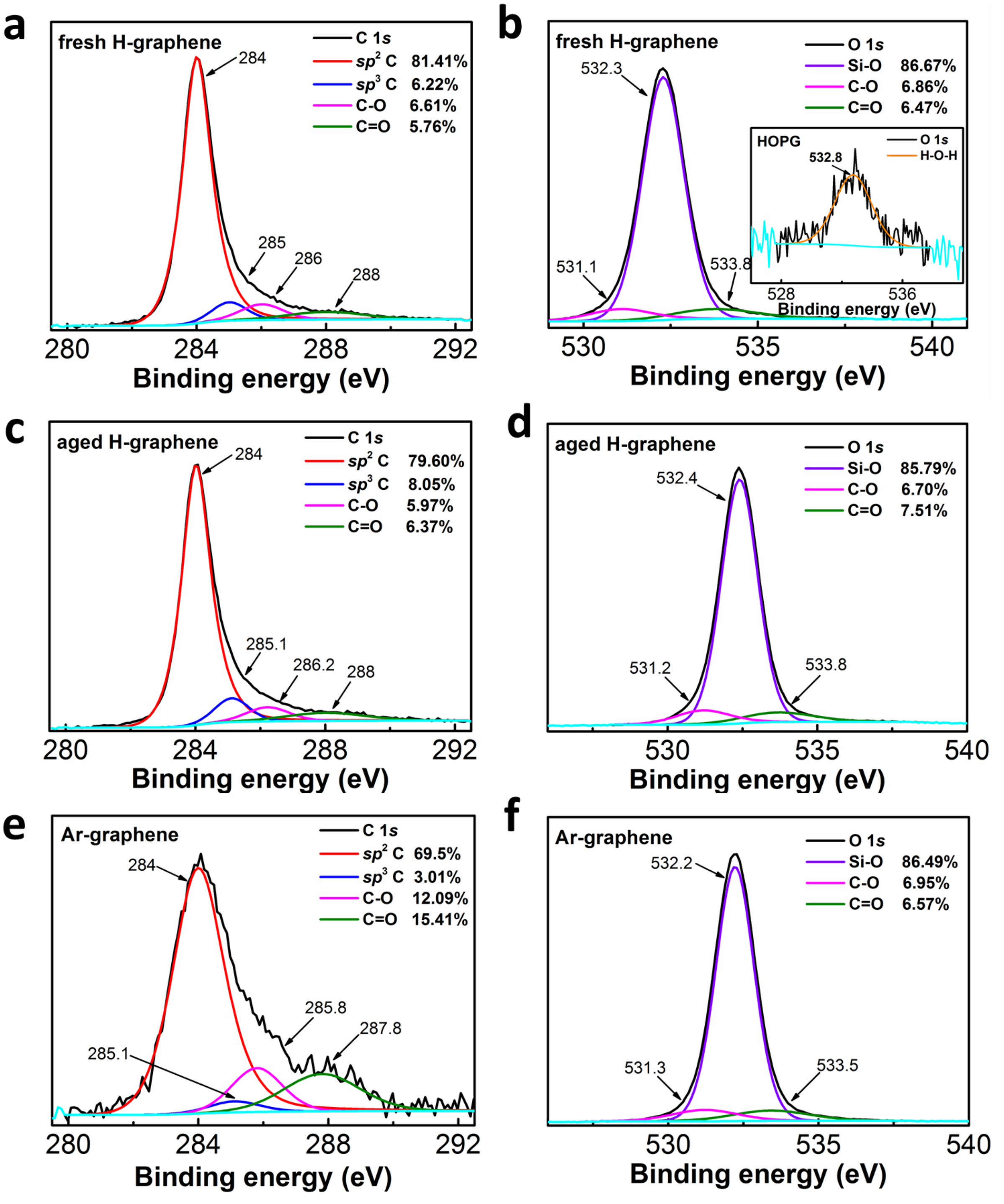

Supplementary Figure 8 │ XPS analysis of H-graphene (60 s) and Ar-graphene (13 s) on a Si/SiO_2 substrate. a-b, C 1*s* (**a**) and O 1*s* (**b**) spectra of H-graphene in fresh state. Inset in **b**) is the O 1*s* spectrum for fresh HOPG. **c-d,** C 1*s* (**c**) and O 1*s* (**d**) spectra of H-graphene after aging in atmosphere for one week. **e-f,** C 1*s* (**e**) and O 1*s* (**f**) spectra of Ar-graphene in fresh state.

Supplementary Tables

Supplementary Table 1 │ XPS analysis (C 1*s* and O 1*s* peaks) of H- vs Ar-graphene deposited on a Si/SiO_2 wafer

	**C 1*s***			**O 1*s***		
	**BE (eV)**		**at (%)**	**BE (eV)**		**at (%)**
**H-graphene (fresh)**	284	sp^2 C	81.41			
	285	sp^3 C	6.22	532.3	Si-O	86.67
	286	C-O	6.61	533.8	C-O	6.86
	288	C=O	5.76	531.1	C=O	6.47
**H-graphene (aged)**	284	sp^2 C	79.60			
	285	sp^3 C	8.05	532.4	Si-O	85.79
	286	C-O	5.97	533.6	C-O	6.70
	288	C=O	6.37	531.2	C=O	7.51
**Ar-graphene**	284	sp^2 C	69.5			
	285.1	sp^3 C	3.01	532.2	Si-O	86.49
	285.8	C-O	12.09	533.5	C-O	6.95
	287.8	C=O	15.41	531.3	C=O	6.57

*aged: one week after sample preparation.

BE: binding energy

Supplementary Notes

Supplementary Note 1: Raman characterization of exfoliated and CVD graphene

To better identify the density and the nature of H-sp^3 defects generated through hydrogenation, in this study we conduct Raman mapping on CVD graphene on Si/SiO_2 (Supplementary Figure 1a) and on exfoliated graphene (Supplementary Figure 1b). Moreover, Supplementary Figure 2a shows the Raman spectra acquired on both exfoliated graphene on Si/SiO_2 (left panel) and CVD graphene on photopolymer (right panel), prepared with minimized contaminations (i.e. no PMMA residues). The signal-to-noise (SNR) ratio is lower in case of CVDG on polymer compared to graphene on Si/SiO_2 because: i) a low laser power is required to prevent heating of the underlying delicate polymer substrate; ii) the redundant peaks from the polymer (at ~1432 cm^{-1} and ~1760 cm^{-1}) render the Raman measurements on CVDG more difficult. Supplementary Figure 2b shows the peak intensities and the I(D)/I(G) ratio for exfoliated and CVD graphene after 2 s, 5 s and 10 s of hydrogenation. The Raman data for both types of graphene are comparable, suggesting similar quality (i.e. H_2-plasma induced defects) for the two types of graphene.

The defect density n_{D} of H-sp^3 defects in graphene can be estimated based on I(D)/I(G) using Supplementary Equation 1: [1]

$$n_{\mathrm{D}} = \frac{(1.8 \pm 0.5) \times 10^{22} (I_{\mathrm{D}}/I_{\mathrm{G}})}{\lambda_{\mathrm{L}}^4} \qquad (1)$$

where λ_{L} denotes the excitation laser wavelength, which is 532 nm here. Moreover, the average distance between defects sites, L_{D} (nm), can be calculated based on $n_{\mathrm{D}}(\mathrm{cm}^{-2}) = 10^{14}/(\pi L_{\mathrm{D}}^2)$.

Supplementary Note 2: Quantum capacitance calculation

In detail, quantum capacitance (C_{q}) is measured by adopting a two-electrode configuration (Supplementary Figure 4). The minimum quantum capacitance, $C_{\mathrm{q,min}}$, is related to the additional carrier density n^* by the following Supplementary Equation 2

$$C_{\mathrm{q}} = \frac{2e^2\sqrt{n_{\mathrm{G}} + n^*}}{\hbar v_{\mathrm{F}}\sqrt{\pi}} \qquad (2)$$

where $n_{\mathrm{G}} = (\frac{eV_{\mathrm{ch}}}{\hbar v_{\mathrm{F}}\sqrt{\pi}})^2$ represents the carrier density created by the gate voltage (V_{g}).[2] Then we extracted the n^* from $C_{\mathrm{q,min}}$ when $n_{\mathrm{G}} = 0$.

Furthermore, based on self-consistent theory[3], we determine the impurity density, n_{imp}, based on n^*

$$n_{\mathrm{imp}} = \frac{n^*}{[2r_{\mathrm{s}}^2 C_0^{\mathrm{RPA}}\left(r_{\mathrm{s}}, a = 4d\sqrt{\pi n^*}\right)]} \qquad (3)$$

where $r_{\mathrm{s}} = \frac{2e^2}{\hbar v_{\mathrm{F}}(\epsilon_1+\epsilon_2)}$, C_0^{RPA} is the correlation function from the random phase approximation (RPA), and d (~1 nm) is the average distance between the charged impurity and graphene. ϵ_1 is the dielectric constant of the photopolymer and ϵ_2 the dielectric constant of electrolyte solution, respectively.

Supplementary Note 3: $\boldsymbol{k^0}$ calculation

We used the Nicholson's method[4] to calculated k^0 with Supplementary Equation 4

$$\psi = k^0 (D_\mathrm{O}/D_\mathrm{R})^{\alpha/2} (RT/\pi nFD_\mathrm{O}\, v)^{1/2} \qquad (4)$$

where ψ is a dimensionless kinetic parameter determined by ΔE_p,

$$\psi = \frac{(-0.6288 + 0.00241\, n\, \Delta E_\mathrm{p})}{(1 - 0.017 n \Delta E_\mathrm{p})} \qquad (5)$$

α is the transfer coefficient, n is the number of electrons transferred, F is the Faraday constant (96500 C mol^{-1}), D_O and D_R is the diffusion coefficient of redox molecules ($\mathrm{cm^2\ s^{-1}}$), v is the scan rate ($\mathrm{V\ s^{-1}}$), R is the gas constant ($\mathrm{VCK^{-1}\ mol^{-1}}$), and T means the temperature in K. Basically, the diffusion coefficients of the reduced and oxidized form for the redox probe are regarded as approximately equal, which gives α about 0.5. As a result, we can apply the Nicholson's method in a simplified form to determine k^0,

$$\psi = k^0 \sqrt{RT/\pi nFDv} \qquad (6)$$

Additionally, the diffusion coefficient, D, can be determined by Randles–Sevcik equation

$$i_\mathrm{p} = 0.4463\, nFAC \sqrt{\frac{nFDv}{RT}} \qquad (7)$$

where A is the effective graphene surfaces that were in contact with electrolyte solution.

Supplementary Note 4: Reproducibility

We reproduced the hydrogenation on several CVDG samples which all showed at least around 2-times increase in both the CV current density (Supplementary Figure 5a) and k^0 (Supplementary Figure 5b) after 1 s of hydrogenation. The differences between k^0 on untreated graphene can be ascribed to the well-known sample-to-sample variations between CVDG originating from the defects formation during the growth or fabrication, or even airborne contaminations from the environment. In addition, the peak-to-peak separation (ΔE_p) for the redox peaks in Supplementary Figure 5c has a minimum after 1 s of hydrogenation, indicating a more reversible electrochemical process on H-graphene (1 s) comparing to untreated graphene and other H-graphene with longer times of hydrogenation treatments.

Supplementary Note 5: Averaged total capacitance

In Supplementary Figure 6, we performed capacitive cyclic voltammetry (CV) in 0.1 M KCl solution containing 10 mM Tris on hydrogenated graphene to understand the impact of hydrogenation on the total capacitance. The capacitive current/current density varies with the scan rate (Supplementary Figure 6a), and can be unified using Supplementary Equation 8:

$i\ (\mathrm{A}) = C(\mathrm{F}) \times v\ (\mathrm{V\ s^{-1}})$ or $j\ (\mathrm{A\ cm^{-2}}\ ) = C(\mathrm{F\ cm^{-2}}) \times v\ (\mathrm{V\ s^{-1}})$. (8)

We extracted the specific capacitance $C_{\mathrm{ave-tot}}$ of graphene based on the linearity between the current densities and scan rates. For example, the $C_{\mathrm{ave-tot}}$ calculated at the potential of –0.25 V is around 5.37 μF cm^{-2} (Supplementary Figure 6a and b). Consistent with the trend of k^0 versus hydrogenation, the $C_{\mathrm{ave-tot}}$ (extracted from the CVs in Supplementary Figure 6c) also increases dramatically after 1 s, and then decreases (2-10 s) and stabilizes at the level of 7.3 μF cm^{-2} till 30 s.

Supplementary Note 6: Raman and electrochemical characterization of Ar treated graphene

To understand to what extend the nature of the defect impacts the electrochemical activity of graphene, we studied vacancy defects on CVD graphene introduced by Ar plasma (Ar-graphene) with comparable defect density levels as for hydrogenated graphene. Supplementary Figure 7a,b demonstrates the comparable densities (i.e. I(D)/I(G)) of Ar-graphene with hydrogenated ones in Figure 1. The electrochemical measurements on Ar-graphene in Figure 3e and f illustrate the barely varied current density and electron transfer rate under the studied defect density. The result echoes well with previous report[5,6] where low densities of vacancy defect did not affect the electrochemical activity of the graphene basal plane. However, a dramatically higher density of vacancy defects is expected to improve the electrochemical kinetics at the cost of a lower in-plane electron transport.[7]

Supplementary Note 7: XPS of H-graphene and Ar-graphene

X-ray photoelectron spectroscopy (XPS) was performed on CVD graphene after hydrogenation and Ar plasma treatment to characterize the chemical stability of plasma treated graphene in ambient condition, and to determine the origin of the H-sp^3 contributions in H-graphene. An asymmetrical fitting function[8] in combination with Gaussian-Lorentzian functions was used to fit the C 1*s* spectra. Other symmetric components were fitted with a Gaussian-Lorentzian fitting function. Shirley background was subtracted for the fitting.

On both fresh and aged (one week) samples (Supplementary Figure 8a and c), the C 1*s* peak of H-graphene can be deconvoluted into four main peaks: C-C sp^2 (284 eV), C-C sp^3 (285 eV), C-O (286-286.2 eV) and C=O (288 eV).[9] Confirmed by the atomic ratio analysis (in %) in Supplementary Table 1, the chemical compositions in H-graphene were stable over time, suggesting a negligible oxidation of H-graphene after aging in ambient conditions. The slight decrease in sp^2/sp^3 ratio for aged samples could originate from the further adsorption of airborne hydrocarbon contaminants. Furthermore, the O 1*s* spectra in Supplementary Figure 8b (fresh) and d (aged) also agree well with 1:1 ratio between single and double bonded oxygen groups in H-graphene, except for the dominant Si-O component (from the Si substrate). Consequently, the detected oxygen bands are likely to be attributed to the residues of polymer (PMMA) employed for the graphene transfer process (PMMA was only used to prepare the XPS samples). In fact, 1) highly ordered pyrolytic graphite (HOPG) was not exposed to PMMA and did not show the O 1*s* band (inset in Supplementary Figure 8b), and 2) Ar-graphene (Supplementary Figure 8e and f), which was also exposed to PMMA, showed similar oxygen bands as for H-graphene.

Furthermore, we compare the C 1*s* peak of graphene containing similar defect densities after 60 s hydrogenation (Supplementary Figure 8a) and after 15 s of Ar plasma treatment (Supplementary Figure 8e). In addition to the existence of C–O and C=O components, ~3% sp^3 C component was found in Ar-graphene, which is probably attributed to the surface contaminations (i.e. PMMA residues and hydrocarbons adsorption).

Supplementary References

1 Cançado, L. G. *et al.* Quantifying defects in graphene via Raman spectroscopy at different excitation energies. *Nano Lett.* **11**, 3190-3196 (2011).

2 Das, A. *et al.* Monitoring dopants by Raman scattering in an electrochemically top-gated graphene transistor. *Nat. Nanotech.* **3**, 210-215 (2008).

3 Adam, S., Hwang, E., Galitski, V. & Sarma, S. D. A self-consistent theory for graphene transport. *Proc. Natl. Acad. Sci.* **104**, 18392-18397 (2007).

4 Nicholson, R. S. Theory and application of cyclic voltammetry for measurement of electrode reaction kinetics. *Anal. Chem.* **37**, 1351-1355 (1965).

5 Velický, M. *et al.* Electron transfer kinetics on natural crystals of MoS_2 and graphite. *Phys. Chem. Chem. Phy.* **17**, 17844-17853 (2015).

6 Zhong, J.-H. *et al.* Quantitative correlation between defect density and heterogeneous electron transfer rate of single layer graphene. *J. Am. Chem. Soc.* **136**, 16609-16617 (2014).

7 Vicarelli, L., Heerema, S. J., Dekker, C. & Zandbergen, H. W. Controlling defects in graphene for optimizing the electrical properties of graphene nanodevices. *ACS Nano* **9**, 3428-3435 (2015).

8 Wertheim, G. Deconvolution and smoothing: applications in ESCA. *J. Electron Spectrosc. Relat. Phenom.* **6**, 239-251 (1975).

9 Ganguly, A., Sharma, S., Papakonstantinou, P. & Hamilton, J. Probing the thermal deoxygenation of graphene oxide using high-resolution in situ X-ray-based spectroscopies. *J. Phys. Chem. C* **115**, 17009-17019 (2011).
